# The role of loneliness and self-concept clarity in the relationship between problematic mobile social network usage and social anxiety among college students

**DOI:** 10.3389/fpsyg.2025.1600474

**Published:** 2025-12-09

**Authors:** Hao Fang, Xiaoyu Xu, Shuyi Yang

**Affiliations:** Zhejiang Agricultural Business College, Shaoxing, China

**Keywords:** problematic mobile social network usage, social anxiety, loneliness, self-concept clarity, college students

## Abstract

**Background:**

Previous studies demonstrated a correlation between problematic mobile social network usage and social anxiety among college students, but the mechanisms of the relationship have not been fully understood.

**Objective:**

The present study aims to examine the mediating role of loneliness and the moderating effect of self-concept clarity in the relationship between problematic mobile social network usage and social anxiety among college students.

**Methods:**

A total of 1,021 college students (mean age = 18.06, SD = 0.26) completed the UCLA Loneliness Scale, Interaction Anxiousness Scale, Problematic Mobile Social Network Usage Scale, and Self-concept Clarity Scale.

**Results:**

Results confirmed that problematic mobile social network usage significantly predicts social anxiety. Loneliness mediated this relationship, and self-concept clarity significantly moderated the mediation process.

**Conclusion:**

These findings underscore the importance of addressing problematic mobile social network usage and loneliness in interventions aimed at reducing social anxiety among college students.

## Introduction

Social anxiety, the fear of negative evaluation in social situations, is a common concern among college students and might significantly affect their academic and social experiences (Rapee and Heimberg, 1997). In China, research by Zhao and Dai (2016) indicated that Chinese college students exhibited significantly higher average levels of social anxiety. A study involving 820 participants revealed that 29.65% of college students had experienced high levels of social anxiety (Zhang et al., 2020). Among Chinese college students, social anxiety manifested uniquely through avoidance of group discussions and reluctance to seek professional help (Zha et al., 2023). These behaviors exacerbated academic underachievement. While mobile social networks allowed for interpersonal communication without the immediate pressure of in-person interactions, the fear of being judged online might have exacerbated social anxiety (Hofmann, 2007; She et al., 2023). Given its pervasive impact, identifying modifiable risk factors is critical for intervention.

In the digital era, mobile social networks are integrated into college students’ social lives as an essential component, providing platforms that enable communication, offer entertainment and allow for self-expression (Andreassen et al., 2017; Shannon et al., 2022; Tandoc et al., 2015). Although these technologies facilitate connection and engagement, they bring about risks, particularly regarding problematic usage. Problematic mobile social network usage is typified by an inordinate preoccupation with mobile social networks, advancing to a level where it affects an individual’s daily activities and undermines their wellbeing (González-Nuevo et al., 2023; Wang et al., 2024; Young, 1998; Young et al., 2017). This phenomenon is of particular concern among college students.

There is increasing attention on the problematic mobile social network usage among college students, concentrating on exploring the psychological mechanisms underlying such behavior. The association between problematic mobile social network usage and various negative outcomes have been widely reported, including increased anxiety, depression and academic impairment (Hussain and Griffiths, 2021; Peng et al., 2006; Yigiter et al., 2024). problematic mobile social network usage has been identified as a predictor of social anxiety (Caplan, 2002; Weidman et al., 2012; Lee and Stapinski, 2012). A study involving 1,082 Chinese college students found that problematic mobile social network usage was significantly positively correlated with online social anxiety, and that problematic mobile social network usage could significantly predict online social anxiety (Liu et al., 2022). While previous studies have explored direct links between social media use and anxiety, few have examined the internal psychological processes.

Loneliness, conceptualized as a subjective sense of social isolation, may play a pivotal role in the relationship between problematic mobile social network usage and social anxiety. According to Social Compensation Theory (Caplan, 2005), individuals experiencing social difficulties may engage in excessive mobile social network usage as a compensatory strategy. However, this heightened online interaction often displaces face-to-face communication, diminishes the quality of real-world social connections, and consequently intensifies feelings of loneliness (Lee and Lee, 2023; O’Day and Heimberg, 2021; Wang and Zeng, 2024). The Cognitive-Behavioral Model of Social Anxiety (Rapee and Heimberg, 1997) posits that elevated loneliness reinforces negative self-perceptions and maladaptive cognitions (e.g., “others will reject me”), leading to increased avoidance behaviors and exacerbated social anxiety (Lim et al., 2016; Federman et al., 2024). Therefore, we propose that loneliness may mediate the relationship between problematic mobile social network usage and social anxiety.

However, empirical findings on the mediating role of loneliness between problematic mobile social network usage and social anxiety are inconsistent. While some studies reported significant indirect effects of problematic mobile social network usage on social anxiety through loneliness (Liu et al., 2022; She et al., 2023), replication of these results has not been achieved in other research (Kim et al., 2019). This inconsistency suggests the potential influence of moderating variables, e.g., self-concept clarity. Self-concept clarity, defined as the extent to which an individual’s self-beliefs are clear, internally consistent and temporally stable (Campbell et al., 1996), has been examined in various studies. Current reports present divergent perspectives regarding the role of self-concept clarity. On one hand, individuals with high self-concept clarity possess more stable and coherent self-views, typically demonstrating greater resilience against stressors and potentially increased resistance to the appeal of problematic mobile social network usage (Lodi-Smith and Roberts, 2010; Parise et al., 2019), owing to their reduced reliance on external validation (Lodi-Smith and Roberts, 2010). Conversely, emerging evidence suggests that when mobile social network usage becomes problematic, individuals with high self-concept clarity might be more sensitive to the dissonance between their well-defined self-perception and the superficial nature of online interactions, potentially resulting in stronger experiences of loneliness (Pang et al., 2024). Considering these contrasting possibilities, this study aims to investigate whether self-concept clarity moderates the effect of problematic usage on loneliness.

Grounded in the Cognitive-Behavioral Model of Social Anxiety (Rapee and Heimberg, 1997) and Social Compensation Theory (Caplan, 2005), this study establishes a moderated mediation model. This model addresses two primary questions: (1) how loneliness transmits the effect of problematic mobile social network usage on social anxiety, and (2) whether self-concept clarity moderates this mediation process by altering the strength of the pathway from problematic mobile social network usage to loneliness.

### Goals of the study

The present research established and validated a moderated mediation model, with the aim of examining the mediating influence of loneliness in the relationship between problematic mobile social network usage and social anxiety among college students. Additionally, it investigated the moderating impact of self-concept clarity on the mediation process. The following hypotheses were tested:

*Hypotheses 1 (H1): Problematic mobile social network usage significantly predicts social anxiety among college students.*

*Hypotheses 2 (H2): Loneliness significantly and positively mediates the relationship between problematic mobile social network usage and social anxiety.*

*Hypotheses 3 (H3): Self-concept clarity moderates the effect of problematic mobile social network usage on loneliness. Compared to individuals with low self-concept clarity, problematic mobile social network usage is more likely to affect loneliness in individuals with high self-concept clarity.*

## Materials and methods

### Participants

Participants were recruited from full-time undergraduate students in China. All participants were required to be fluent in reading and understanding Chinese, with no history of clinically diagnosed mental disorders. From 10th April 2023 to 27th April 2023, a total of 1,104 participants volunteered to participate in the study through an online survey system. Eighty three participants were excluded due to incomplete questionnaires or missing demographic information. A final sample of 1,021 valid participants (validity rate of 92%) was included in the present study. Among them, 532 were male (52.1%) and 489 were female (47.9%). Their average age was 18.06 (*SD* = 0.26). No incentives were provided for participation.

### Measurement

#### UCLA loneliness scale

The scale used in this study was adopted the UCLA Loneliness Scale by Ip et al. (2024), which includes 20 items measuring both positive (e.g., “Do you often feel like someone truly understands you?”) and negative dimensions (e.g., “Do you often feel lonely?”) of loneliness. The scale scored on a 4-point scale from 1 to 4 (1 = never felt this way, 4 = always felt this way). The higher the score on the scale, the stronger the sense of loneliness. The Cronbach’s alpha used in this study was 0.89.

#### Interaction anxiousness scale

The social anxiety of individuals is measured using the Interaction Anxiousness Scale (IAS) developed by Leary (1983). The IAS consists of 15 items (e.g., “Even at informal gatherings, I often feel nervous”), each scored on a 5-point scale from 1 to 5 (1 = not at all characteristic, 5 = extremely characteristic). A higher score obtained from this scale was associated with a correspondingly higher level of social anxiety in participants. The higher the score on this scale, the higher the level of social anxiety. The Cronbach’s alpha for this study was 0.83.

#### Problematic mobile social network usage scale

The scale used is developed by Liu and Ma (2018). This scale has been extensively evaluated. It consists of 20 items (e.g., “I always unconsciously pick up my phone and open social apps, aimlessly browsing through them”), each scored on a 5-point scale from 1 to 5 (1 = not at all characteristic, 5 = completely characteristic). Higher scores on the scale demonstrated a greater degree of problematic mobile social network use among individuals. The higher the score on the scale, the higher the individual’s problematic use of mobile social networks. The Cronbach’s alpha for this study was 0.96.

#### Self-concept clarity scale

The Self-Concept Clarity Questionnaire, developed by Campbell et al. (1996), was used, which includes three dimensions: clarity, temporal stability and internal consistency, with a total of 12 items (e.g., “I can clearly know who I am and what kind of person I am”), scored on a 5-point scale from 1 to 5 (1 = strongly disagree, 5 = strongly agree). Higher scores on the scale indicated to a greater level of self-concept clarity among participants. The higher the score on the scale, the higher the individual’s level of self-concept clarity. The Cronbach’s alpha for this study was 0.85.

### Data analysis

SPSS 26.0 (IBMCorp., Armonk, N.Y., USA) was used for descriptive statistics and correlation analysis. The mediating and moderating effects were tested using Hayes’PROCESS macro (Model 4 for mediation and Model 7 for moderated mediation). For mediation, we tested whether loneliness mediated the relationship between problematic mobile social network usage and social anxiety. For moderation, we examined whether self-concept clarity moderated the path from problematic mobile social network usage to loneliness. Bootstrap sampling was used to estimate confidence intervals, with gender and grade included as control variables.

## Results

### Common method bias test

For testing common method bias, Harman’s single-factor test was mainly used (Zhou and Long, 2004). Exploratory factor analysis was conducted on all variables included in the four questionnaires. From the results of the analysis, there were 10 factors with eigenvalues greater than one, while the variance explained by the first factor was much less than 40%, at 25.05%. The results suggested that common method bias does not exist.

### Descriptive statistics and bivariate correlations

After correlation analysis around four factors of problematic mobile social network usage, social anxiety, loneliness, and self-concept clarity, it was found that problematic mobile social network usage was positively correlated with loneliness and social anxiety, and negatively correlated with self-concept clarity. Loneliness was negatively correlated with self-concept clarity and positively correlated with social anxiety. Self-concept clarity was significantly and negatively correlated with social anxiety (Table 1).

**Table 1:** Results of descriptive statistics and correlation analysis.

Variables	*M*	*SD*	1	2	3	4	5	6
1 Gender			1					
2 Grade			−0.095**	1				
3 Problematic mobile social network usage	2.95	0.03	0.078*	0.037	1			
4 Loneliness	2.10	0.02	−0.016	0.014	0.330**	1		
5 Self-concept clarity	2.79	0.02	−0.042	0.028	−0.482**	−0.354**	1	
6 Social Anxiety	3.21	0.02	0.163**	−0.015	0.520**	0.419**	−0.444**	1

*N* = 1, 021. Grade and gender are dummy variables, freshman = 1, sophomore = 2, junior = 3, senior (graduation year) = 4; male = 1, female = 2; *p < 0.05, **p < 0.01, ***p < 0.001, same below.

### Mediating effects with moderation

We used Model 4 of the PROCESS macro from SPSS with 5,000 bootstrapped samples and included gender and grade as control variables to evaluate the negative relationship between problematic mobile social network usage and social anxiety and the mediating role of loneliness. Results indicated that problematic mobile social network usage significantly and positively predicted social anxiety (b = 0.293, *p* < 0.001). Problematic mobile social network usage significantly and positively predicted loneliness (*b* = 0.195, *p* < 0.001). Loneliness significantly and positively predicted social anxiety (*b* = 0.340, *p* < 0.001). Hypothesis 1 was therefore supported.

**Figure 1:**
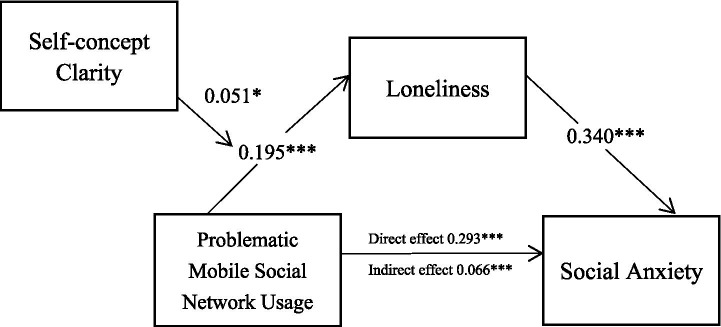
Mediating effects of loneliness.

The mediating effect of loneliness was statistically significant (*b* = 0.066, SE = 0.010, 95% CI [0.048, 0.086]). The 95% confidence interval did not include zero, indicating that loneliness was a significant mediator between problematic mobile social network usage and social anxiety. The mediating effect accounted for 18.50% of the total effect (see Figure 1). Hypothesis 2 was therefore supported.

**Figure 2:**
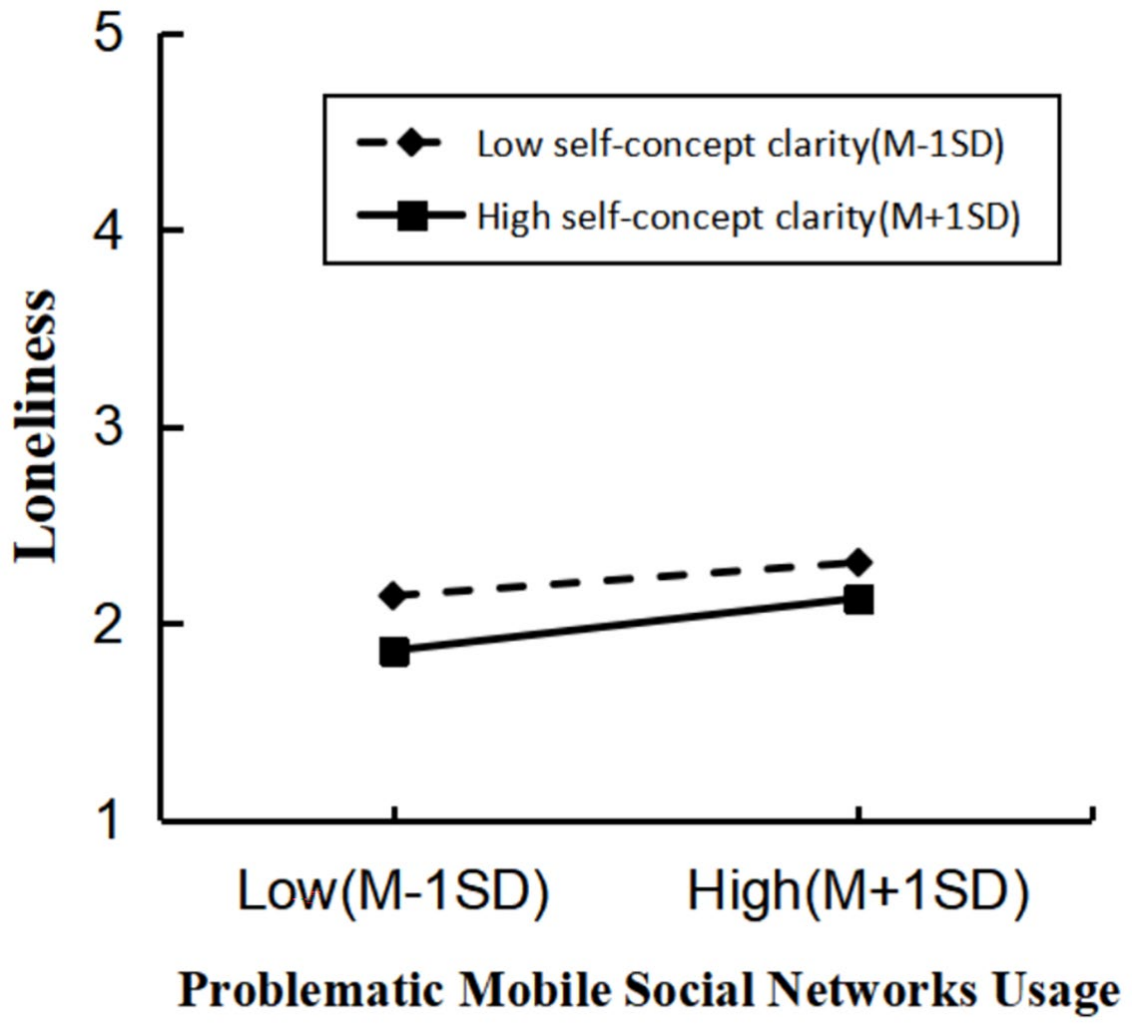
Moderating effect of self-concept clarity in problematic mobile social network usage and loneliness.

We used Model 7 in Hayes’PROCESS macro to test the moderating effect of self-concept clarity on the mediation pathway. The interaction between problematic mobile social network usage and self-concept clarity significantly predicted loneliness (*b* = 0.051, *p* < 0.05). Simple slope analysis (Figure 2) showed that problematic mobile social network usage positively predicted loneliness at both high (+1 SD) and low (–1 SD) levels of self-concept clarity, and the effect was stronger at high self-concept clarity (simple slope _high_ = 0.160, *p* < 0.001; simple slope _low_ = 0.102, *p* < 0.001).

We also examined whether self-concept clarity moderated the indirect effect of loneliness. Such indirect effect was significant at both high and low self-concept clarity, but the magnitude differed. At low self-concept clarity, indirect effect was equal to 0.035 (95% CI [0.018, 0.053]). At high self-concept clarity, indirect effect was equal to 0.054 (95% CI [0.034, 0.076]). The difference between these indirect effects was significant (difference = 0.017, 95% CI [0.001, 0.034]), indicating that regardless of whether individuals have high or low self-concept clarity, problematic mobile social network usage leads to social anxiety through the mediating role of loneliness. For individuals with higher self-concept clarity, the indirect effect of problematic mobile social network usage on social anxiety through loneliness is stronger. Hypothesis 3 was therefore supported.

## Discussion

The present study investigated the complex relationships among problematic mobile social network usage, social anxiety, loneliness and self-concept clarity among college students, as well as a moderated mediation model in which loneliness mediates the relationship between problematic mobile social network usage and social anxiety, with self-concept clarity moderating this mediation process.

Both loneliness and social anxiety were positively associated with problematic mobile social network usage. However, a negative correlation was detected between problematic mobile social network usage and self-concept clarity. These findings were consistent with previous research, suggesting that individuals suffering from higher levels of social anxiety and loneliness were more likely to engage in problematic mobile social network usage as a coping mechanism (Caplan, 2002; Ge et al., 2025; O’Day and Heimberg, 2021; Weidman et al., 2012). Both loneliness and social anxiety were positively associated with problematic mobile social network usage, while self-concept clarity was negatively correlated with such usage. This aligns with previous research, suggesting that individuals with unclear self-concepts may rely more on external validation from social media, leading to problematic usage patterns (Lodi-Smith and Roberts, 2010).

The mediating role of loneliness in the relationship between problematic mobile social network usage and social anxiety has been confirmed through mediation analysis. Consistent with the Cognitive-Behavioral Model (Rapee and Heimberg, 1997), the results indicated that loneliness, as a form of negative self-perception, exacerbated social anxiety. Loneliness significantly mediated the relationship, accounting for a substantial proportion of 18.50% of the total effect. The observation suggested that college students have problems using mobile social networks, which might increase loneliness and exacerbate their social anxiety. The finding was consistent with the Social Compensation Theory (Caplan, 2002), which suggested that individuals who had difficulties in real-world social interactions tend to over-rely on mobile social networks for compensation. Problematic mobile social network usage reduces offline socializing, weakens genuine emotional connections, and thereby intensifies loneliness, ultimately leading to increased social anxiety.

The moderation analysis results supported our research hypothesis, indicating that self-concept clarity plays a moderating role in the relationship between problematic mobile social network usage and loneliness. Specifically, compared to individuals with low self-concept clarity, problematic mobile social network usage had a more pronounced impact on loneliness among those with high self-concept clarity. This finding aligns with studies suggesting that individuals with high self-concept clarity have a heightened awareness regarding the gap between their social needs and actual social experiences (Pang et al., 2024). When these individuals engage in excessive use of mobile social networks, they become more acutely aware of the negative impact of excessive social media use on their well-being and recognize that online interactions fail to fulfill their deeper social needs, thereby experiencing more intense feelings of loneliness (Lewandowski et al., 2010). For example, a student with a clear self-view as “sociable” might feel lonelier if problematic scrolling through social media highlights a lack of deep, meaningful interactions compared to their self-perception.

This finding appears inconsistent with previous research demonstrating that self-concept clarity generally buffers against negative psychological outcomes (Lodi-Smith and Roberts, 2010; Wong et al., 2019). The discrepancy could be explained by the distinctive nature of problematic mobile social network usage. While prior studies have primarily examined general stressors or normal social media use, in the specific context of problematic usage characterized by loss of control and preoccupation, the self-awareness of individuals with high self-concept clarity may paradoxically heighten their sensitivity to the negative impact of their usage behaviors on social well-being, thereby strengthening its association with loneliness.

These findings hold significant implications for exploring the psychological mechanisms underlying social anxiety among college students. The study emphasizes that individuals’ problematic mobile social network usage and loneliness serve as effective intervention entry points for addressing social anxiety (Du et al., 2024). Intervention strategies such as enhancing social skills, strengthening social support, improving interpersonal interactions, and adjusting negative social cognition can effectively alleviate individuals’ loneliness (Masi et al., 2011). Additionally, fostering self-concept clarity may help college students cope with the complexity of social anxiety in a healthier manner.

## Limitations and future research

The current study used a cross-sectional design method. Longitudinal research will be needed to establish the temporal sequence of the observed relationships and to further explore the potential reciprocal effects among problematic mobile social network usage, loneliness, social anxiety, and self-concept clarity.

## Conclusion and educational suggestions

This study revealed significant insights into the relationship between problematic mobile social network usage and social anxiety among college students, highlighting the mediating role of loneliness and the moderating role of self-concept clarity. The findings indicated that loneliness acts as a mediator, linking excessive use of mobile social networks to increased levels of social anxiety. Additionally, the present study observed the moderating role of self-concept in the relationship.

Educational institutions are encouraged to implement programs aimed at promoting responsible use of mobile social networks. Workshops and seminars can be organized to educate students about the potential negative consequences of excessive use, including increased loneliness and social anxiety. These could include cognitive restructuring sessions to challenge beliefs. Additionally, peer mentoring programs could facilitate offline social skill practice, encouraging students to engage in real-world social activities and hobbies—reducing reliance on mobile social networks for interaction. To further reinforce stable self-perception, institutions might introduce self-concept journaling (Wu et al., 2023), where daily entries help students cultivate healthier self-awareness.

## Data Availability

The raw data supporting the conclusions of this article will be made available by the authors, without undue reservation.
